# Trend of extracorporeal membrane oxygenation support in patients with acute respiratory distress syndrome in South Korea

**DOI:** 10.1038/s41598-022-09230-9

**Published:** 2022-03-28

**Authors:** Tak Kyu Oh, In-Ae Song

**Affiliations:** 1 Department of Anesthesiology and Pain Medicine, Seoul National University Bundang Hospital, Gumi-ro, 173, Beon-gil, Bundang-gu, Seongnam, 13620 South Korea; 2 Department of Anesthesiology and Pain Medicine, College of Medicine, Seoul National University, Seoul, South Korea

**Keywords:** Anatomy, Health care, Neurology

## Abstract

We examined and compared the clinical characteristics of acute respiratory distress syndrome (ARDS) patients who received and did not receive extracorporeal membrane oxygenation (ECMO) support. The national health insurance database of South Korea was used to obtain real-world data. All adult patients admitted to intensive care units for ARDS treatment between 1 January 2014 and 31 December 2019 were included in this study. Of the 10,173 patients with ARDS included in the analysis, 740 (7.3%) received ECMO support for a mean duration of 1.6 days (standard deviation [SD]: 2.8 days) and were assigned to the ECMO group. The ECMO group had a significantly lower mean age at 57.0 years (SD: 15.7 years) than the non-ECMO group (71.8 Â years [SD: 15.1 Â years], *P* < 0.001). In multivariable logistic regression, a 1-year increase in age was associated with a 5% lower prevalence of ECMO support. The annual case volume was classified into four groups by quartile ratio (Q1 [lowest], Q2, Q3, and Q4 [highest]), and Q2, Q3, and Q4 groups showed a higher prevalence of ECMO support than the Q1 group. ECMO support was also performed more frequently in high case volume centers than in low case volume centers for ARDS patients.

## Introduction

Extracorporeal membrane oxygenation (ECMO) is a therapeutic option that provides artificial support for patients with refractory cardiac and/or respiratory failure in the intensive care unit (ICU)^1^. Acute respiratory distress syndrome (ARDS), a severe condition occurring in the ICU, can be treated with ECMO support as a rescue therapy option^2,3^. ECMO support for ARDS as rescue therapy has increased since the 2009 influenza pandemic^4^. More recently, since the coronavirus disease (COVID-19) pandemic from 2020 until now^5^, ECMO support for COVID-19-associated ARDS has increased globally^6–8^.

There are several considerations when providing ECMO support to patients with ARDS. First, ECMO support is expensive, and the mean estimated cost for ECMO procedures was reported to be 73,112 United States Dollars (USD) in Norway^9^. Moreover, many physicians may encounter sensitive and complex ethical issues regarding the application of ECMO support, such as the meaning and nature of resuscitation, and they do not resuscitate in many cases^10^. For example, patients for whom ECMO support is not indicated, the clinical usefulness, and ethical considerations are important issues^11^. Therefore, a recent ECMO resource planning initiative during the COVID-19 pandemic suggested that age > 65 years or active malignancy, irreversible neurologic injury, and expected life expectancy < 6 months were absolute contraindications for the initiation of ECMO support^12^. However, the trends of clinical applications using real-world data have not been evaluated using a national database. We hypothesized that multiple factors might affect the application of ECMO support in patients with ARDS.

Therefore, using the national health claims’ database in South Korea, we aimed to examine and compare the clinical characteristics of patients with ARDS who underwent ECMO support with those with ARDS who did not receive ECMO support. In addition, the overall survival time and factors associated with ECMO support were evaluated.

## Methods

### Ethical statement, study design, and data source

This was a population-based cohort study based on nationwide settings in South Korea. The Institutional Review Board (IRB) of Seoul National University Bundang Hospital approved the protocol of this study (X-2008-630-903), and the National Health Insurance Service (NHIS) also approved the study protocol (NHIS-2021-1-424). In addition, this study was performed in accordance with the Declaration of Helsinki. The requirement for informed consent was waived by the IRB and NHIS because our study used anonymized data for analysis. We used the NHIS database for this study, which contains physical, socioeconomic, disease diagnosis, and treatment information of individuals in South Korea. As the sole public health insurance system, all disease diagnoses must be registered in the NHIS database using International Statistical Classification of Diseases and Related Health Problems-tenth revision (ICD-10) codes. Moreover, prescription information regarding any procedures and/or drugs should be registered in the NHIS database for patients to receive financial support from the government. The dates of death of the study population were also extracted and used for this study until 31 December 2020. South Koreans pay a fixed rate for health insurance premiums based on their income, with approximately 67% of their medical expenses being subsidized by the government^13^. However, those who cannot afford insurance premiums or have difficulty financially supporting themselves are included in the Medical Aid Program, which involves government support for almost all medical expenses.

### Study population

All adult patients who were ≥ 18 years old and admitted to the ICU between 1 January 2014 and 31 December 2019 with a diagnosis of ARDS (J80) were included in the study. After the consensus on the Berlin Definition of ARDS in 2012^14^, ARDS was diagnosed according to respiratory failure due to acute hypoxemia, dyspnea, and increased bilateral pulmonary infiltration in South Korea. Since ARDS is a pathologic condition that can occur due to other pathologic conditions^14^, we included both cases regarding the main diagnoses and secondary diagnosis of ARDS in this study. Therefore, patients with a primary diagnosis of pneumonia and a secondary diagnosis of ARDS were included in this study. The main diagnosis was defined by the NHIS after the end of hospitalization as the disease wherein the patient’s demand for treatment or examination was the greatest during their hospitalization. If a patient was admitted to the ICU twice or more with a diagnosis of ARDS, only the first episode of ICU admission was considered in this study. Among patients with ARDS in this study, patients who received ECMO support were considered as the ECMO group, while the other patients were considered as the non-ECMO group.

### Study objectives

This study had three primary objectives. First, the clinical characteristics between the ECMO and non-ECMO groups were compared to examine any trend in ECMO application among patients with ARDS. Second, the overall survival times in the ECMO and non-ECMO groups were compared in patients with ARDS. Third, the factors associated with ECMO application in patients with ARDS were investigated.

### Collected variables

The following variables were collected for this study: Physical variables (age and sex) were included, and the annual income level at the time of ARDS treatment was collected to reflect the socioeconomic status of patients with ARDS. The annual income level was registered in the NHIS database to determine insurance premiums, and it was classified into four groups using quartiles. The treatment results of patients with ARDS were collected and categorized into four groups namely, discharge and follow-up in the same hospital group, transfer to other long-term facility center group, discharge and outpatient clinic follow-up group, and the death within hospitalization group. The admission department was listed and classified into two groups as follows: internal medicine and non-internal medicine. Length of hospitalization (days), total cost of hospitalization (USD), and cost per day (USD) were collected. The type of hospital admission was classified into three groups (transfer from another hospital, admission through the emergency room, and admission through outpatient clinic). The annual case volume of ARDS’ admission in the facility was calculated, and patients with ARDS were classified into four groups using quartiles, based on the hospital wherein they were hospitalized (Q1 ≤ 4, Q2: 5–14, Q3: 15–28, and Q4 ≥ 29). The main diagnosis of ARDS, as well as diagnoses of shock (R57) or sepsis (A40, A41, and R65.2), were collected. If patients with ARDS had a main diagnosis of sepsis, the patient was considered to have sepsis-associated ARDS. Information on the treatment of ARDS, including ECMO support, use of neuromuscular blockade (NMB), continuous renal replacement (CRRT), duration of mechanical ventilation (in days), and administration of cardiopulmonary resuscitation (CPR), were collected. To reflect the comorbid status of patients with ARDS, the Charlson comorbidity index (CCI) was calculated using the registered ICD-10 codes of underlying individual diseases (Table S1).

### Statistical methods

The clinicopathological characteristics of ARDS patients are presented as mean values (standard deviation [SD]) for continuous variables and number (%) for categorical variables. To compare clinicopathological characteristics between the ECMO and non-ECMO groups, the t-test and chi-square test were used for continuous variables and categorical variables, respectively. Kaplan–Meier curves were used to present overall survival times among the ECMO and non-ECMO groups. The median survival time with the 95% confidence intervals (CI) between the two groups was compared using the log-rank test. Finally, a multivariable logistic regression model was used to examine the factors associated with the application of ECMO support among all patients with ARDS. All variables were included in the model, and there was no multicollinearity between variables with the criterion of variance inflation factors < 2.0. The results of the logistic regression analyses were presented as odds ratios (ORs) with 95% CIs, and the Hosmer–Lemeshow test was used to confirm the goodness of fit in the multivariable model. All statistical analyses were performed using R software (version 4.0.3, R packages, R Project for Statistical Computing, Vienna, Austria), and statistical significance was set at *P* < 0.05.

## Results

**Figure 1:**
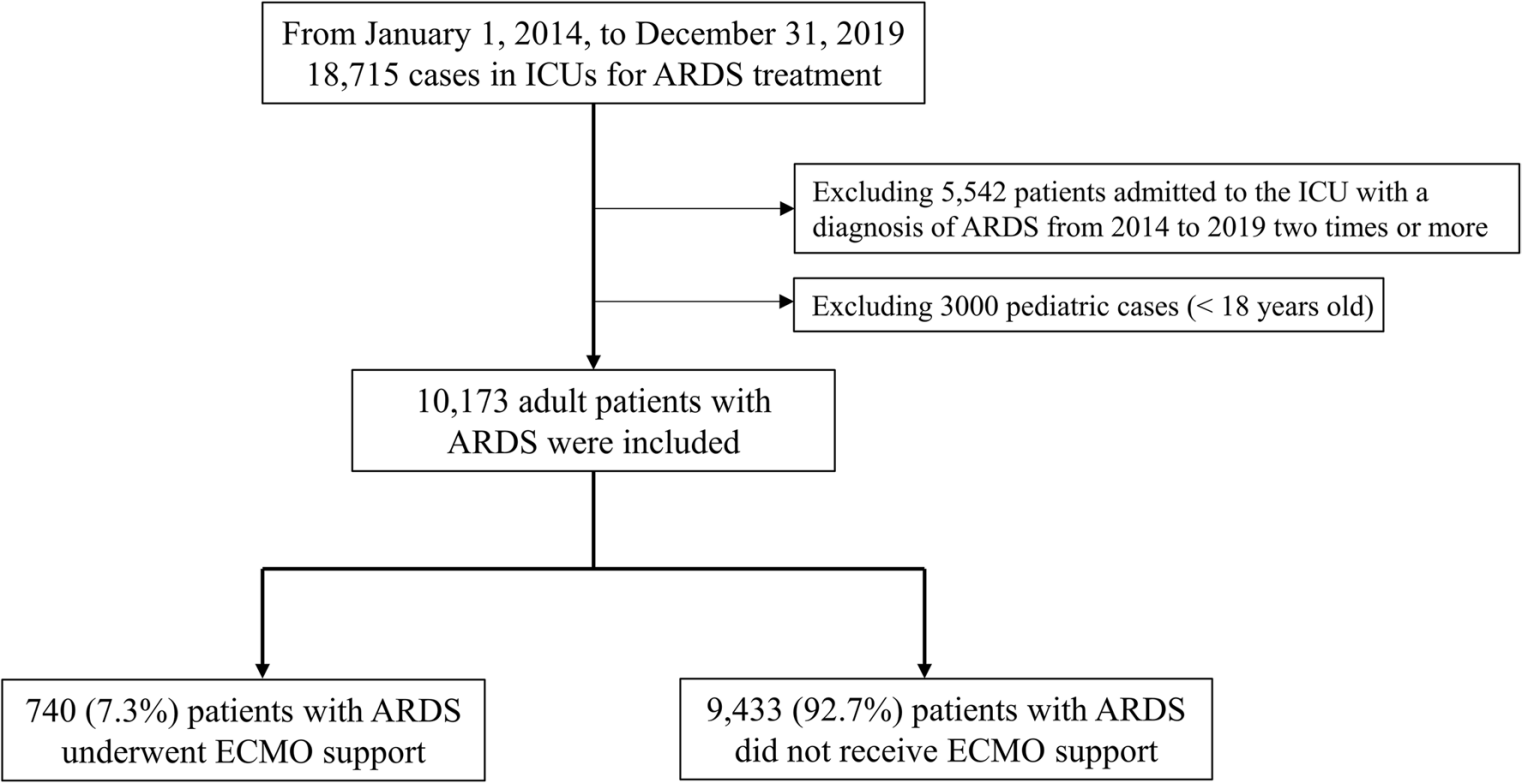
Flow chart depicting the selection process of patients with ARDS patient. ARDS. Acute respiratory distress syndrome.

From 1 January 2014 to 31 December 2019, a total of 18,715 cases of ICU admissions with ARDS’ diagnosis were initially extracted. Among them, 5,542 patients admitted to the ICU twice or more with a diagnosis of ARDS and 3000 pediatric cases (< 18 years old) were excluded from the analysis. Finally, 10,173 adult patients with ARDS were included in the analysis. Among them, a total of 740 (7.3%) patients received ECMO support for a mean duration of 1.6 days (SD 2.8) and were assigned to the ECMO group (Fig. 1). Table 1 shows the clinicopathological characteristics of all patients with ARDS, and 30-day, 90-day, and 365-day mortality occurred in 4,846 (47.6%), 6,276 (61.7%), and 7,051 (69.3%) patients, respectively, after the ARDS’ diagnosis.

**Table 1:** Clinicopathological characteristics of all ARDS’ patients.

Variable	Mean (SD) or N (%)
Age, year	70.7 (15.6)
Sex, male	6,289 (61.8)
**Annual Income level at ARDS treatment**	
Q1 (lowest)	2,870 (28.2)
Q2	1,527 (15.0)
Q3	2,022 (19.9)
Q4 (Highest)	3,610 (35.5)
Unknown	144 (1.4)
**Treatment result**	
Discharge, and follow up in same hospital	2,732 (26.9)
Transfer to other long-term facility center	627 (6.2)
Discharge, and outpatient clinic follow up	2,680 (26.3)
Death within hospitalization	4,134 (40.6)
**Admitting department**	
IM	8,158 (80.2)
Non-IM	2,015 (19.8)
Length of hospitalization, day	
Total cost for hospitalization, USD	11,585.2 (14,234.2)
Cost per day, USD	827.0 (908.7)
**Hospital admission**	
Transfer from other hospital	440 (4.3)
Admission through Emergency Room	6,279 (61.7)
Admission through outpatient clinic	3,454 (34.0)
**Annual case volume of ARDS admission**	
Q1 ≤ 4	1,896 (18.6)
Q2: 5–14	2,429 (23.9)
Q3: 15–28	3,002 (29.5)
Q4 ≥ 29	2,846 (28.0)
Main diagnosis of ARDS	5,127 (50.4)
Sepsis associated ARDS	1,490 (14.6)
Diagnosis of shock during hospitalization	901 (8.9)
CCI at hospital admission for ARDS	4.7 (3.1)
**ECMO support**	740 (7.3)
Duration of ECMO support, day	1.6 (2.8)
NMB use	3,789 (37.2)
**CRRT use**	1,070 (10.5)
Duration of CRRT use, day	3.3 (5.6)
Duration of Mechanical Ventilator support, day	5.2 (8.3)
Experience of CPR during hospitalization	1,160 (11.4)
30-day mortality	4,846 (47.6%)
90-day mortality	6,276 (61.7%)
365-day mortality	7,051 (69.3%)
**Year of admission for ARDS**	
2014	1,707 (16.8)
2015	1,608 (15.8)
2016	1,869 (18.4)
2017	1,706 (16.8)
2018	1,713 (16.8)
2019	1,570 (15.4)

ARDS, acute respiratory distress syndrome; SD, standard deviation; IM, internal medicine; USD, United States Dollars; ECMO, extracorporeal membrane oxygenation; NMB, neuromusclar blockade; CRRT, continuous renal replacement therapy, CPR, cardiopulmonary resuscitation.

Table 2 shows the results of the comparison of clinicopathological characteristics between the ECMO and non-ECMO groups among patients with ARDS. The mean age of the ECMO group was significantly lower at 57.0 years (SD 15.7) than that in the non-ECMO group (71.8 years (SD 15.1), *P* < 0.001). The total cost of hospitalization was higher in the ECMO group (mean: USD 36,416.9 (SD 23,387.3) than in the non-ECMO group (mean: USD 9,637.2 (SD 11,112.2), *P* < 0.001. The prevalence of NMB (645/740, 87.2%) and CRRT (291/740, 39.3%) use in the ECMO group was higher than that of NMB (3,144/9,433, 33.3%) and CRRT (779/9,433, 8.3%) use in the non-ECMO group, *P* < 0.001.

**Table 2:** Comparison of clinicopathological characteristics between the ECMO and non-ECMO groups among ARDS’ patients.

Variable	ECMO patients n = 740	Non-ECMO patients n = 9,433	*P*-value
Age, year	57.0 (15.7)	71.8 (15.1)	< 0.001
Sex, male	484 (65.4)	5,805 (61.5)	0.037
**Annual Income level**			
Q1 (lowest)	172 (23.2)	2,698 (28.6)	< 0.001
Q2	148 (20.0)	1,379 (14.6)	
Q3	169 (22.8)	1,853 (19.6)	
Q4 (Highest)	240 (32.4)	3,370 (35.7)	
Unknown	11 (1.5)	133 (1.4)	
**Treatment result**			< 0.001
Discharge, and follow up in same hospital	263 (35.5)	2,469 (26.2)	
Transfer to other long-term facility center	18 (2.4)	609 (6.5)	
Discharge, and outpatient clinic follow up	127 (17.2)	2,553 (27.1)	
Death within hospitalization	332 (44.9)	3,802 (40.3)	
Admitting department: IM	506 (68.4)	7,652 (81.1)	< 0.001
Length of hospitalization, day	20.6 (15.1)	15.7 (14.4)	< 0.001
Total cost for hospitalization, USD	36,416.9 (23,387.3)	9,637.2 (11,112.2)	< 0.001
Cost per day, USD	2,369.8 (1,581.1)	701.3 (692.6)	< 0.001
**Hospital admission**			< 0.001
Transfer from other hospital	36 (4.9)	404 (4.3)	
Admission through Emergency Room	524 (70.8)	5,755 (61.0)	
Admission through outpatient clinic	180 (24.3)	3,274 (34.7)	
**Annual case volume**			< 0.001
Q1 ≤ 4	14 (1.9)	1,882 (20.0)	
Q2: 5–14	196 (26.5)	2,233 (23.7)	
Q3: 15–28	230 (31.1)	2,772 (29.4)	
Q4 ≥ 29	300 (40.5)	2,546 (27.0)	
Main diagnosis of ARDS	424 (57.3)	4,703 (49.9)	< 0.001
Sepsis associated ARDS	186 (25.1)	1,304 (13.8)	< 0.001
Diagnosis of shock during hospitalization	111 (15.0)	790 (8.4)	< 0.001
CCI at hospital admission for ARDS	4.2 (2.8)	4.7 (3.1)	< 0.001
NMB use	645 (87.2)	3,144 (33.3)	< 0.001
CRRT use	291 (39.3)	779 (8.3)	< 0.001
Experience of CPR during hospitalization	159 (21.5)	1,001 (10.6)	< 0.001
**Year of admission for ARDS**			< 0.001
2014	84 (11.4)	1,623 (17.2)	
2015	107 (14.5)	1,501 (15.9)	
2016	140 (18.9)	1,729 (18.3)	
2017	117 (15.8)	1,589 (16.8)	
2018	143 (19.3)	1,570 (16.6)	
2019	149 (15.1)	1,421 (15.1)	

Presented as mean values (standard deviation) for continuous variables and number (%) for categorical variables.

ARDS, acute respiratory distress syndrome; ECMO, extracorporeal membrane oxygenation; IM, internal medicine; USD, United States Dollars; NMB, neuromusclar blockade; CRRT, continuous renal replacement therapy, CPR, cardiopulmonary resuscitation.

Table 3 shows the results of the multivariable logistic regression model for ECMO support in patients with ARDS. A one-year increase was associated with a 5% lower prevalence of ECMO support (OR, 0.95; 95% CI: 0.95–0.96; *P* < 0.001). Compared to the Q1 annual income level group, the Q2 group (OR: 1.42, 95% CI: 1.09–1.85; *P* = 0.010), Q3 group (OR: 1.37, 95% CI: 1.07–1.77; *P* = 0.014), and Q4 group (OR: 1.35, 95% CI: 1.06–1.70; *P* = 0.013) showed a high prevalence of ECMO support among patients with ARDS. Compared with the Q1 group of annual case volume, the Q2 group (OR: 6.97, 95% CI: 3.84–12.63; *P* < 0.001), Q3 group (OR: 5.24, 95% CI: 2.90–9.49; *P* < 0.001), and Q4 group (OR: 6.50, 95% CI: 3.60–11.72; *P* < 0.001) showed a high prevalence of ECMO support among patients with ARDS. NMB use (OR: 7.39; 95% CI: 5.83–9.37; *P* < 0.001), duration of CRRT use (OR: 1.13, 95% CI: 1.09–1.16; *P* < 0.001), duration of mechanical ventilation (OR: 1.03, 95% CI: 1.02–1.04; *P* < 0.001), and experience of CPR (OR: 1.70, 95% CI: 1.36–2.13; *P* < 0.001), were associated with a higher prevalence of ECMO support among patients with ARDS.

**Table 3:** Multivariable logistic regression model for ECMO support in patients with ARDS.

Variable	OR (95% CI)	*P*-value
Age, year	0.95 (0.95, 0.96)	< 0.001
Sex, male (vs female)	0.98 (0.82, 1.17)	0.805
**Annual Income level at ARDS treatment**		
Q2 (vs Q1; Lowest)	1.42 (1.09, 1.85)	0.010
Q3 (vs Q1; Lowest)	1.37 (1.07, 1.77)	0.014
Q4 (Highest) (vs Q1; Lowest)	1.35 (1.06, 1.70)	0.013
Unknown (vs Q1; Lowest)	1.12 (0.55, 2.32)	0.752
Admitting department: IM (vs non-IM)	0.40 (0.33, 0.49)	< 0.001
**Hospital admission**		
Transfer from other hospital	1	
Admission through Emergency Room	1.13 (0.73, 1.73)	0.587
Admission through outpatient clinic	1.36 (0.86, 2.14)	0.187
**Annual case volume of ARDS admission**		
Q2: 5–14 (vs Q1 ≤ 4)	6.97 (3.84, 12.63)	< 0.001
Q3: 15–28 (vs Q1 ≤ 4)	5.24 (2.90, 9.49)	< 0.001
Q4 ≥ 28 (vs Q1 ≤ 4)	6.50 (3.60, 11.72)	< 0.001
Main diagnosis of ARDS (vs secondary diagnosis of ARDS)	1.32 (1.11, 1.58)	0.002
Sepsis associated ARDS	1.63 (1.32, 2.01)	< 0.001
Diagnosis of shock during hospitalization	1.50 (1.16, 1.93)	0.002
**CCI at hospital admission for ARDS**		
2–3 (n = 2,722) vs 0–1 (n = 1,463)	1.43 (1.08, 1.88)	0.011
4–5 (n = 2,526) vs 0–1 (n = 1,463)	1.20 (0.89, 1.60)	0.227
6–7 (n = 1,767) vs 0–1 (n = 1,463)	1.07 (0.77, 1.49)	0.675
≥ 8 (n = 1,697) vs 0–1 (n = 1,463)	0.89 (0.63, 1.25)	0.504
NMBA use	7.39 (5.83, 9.37)	< 0.001
Duration of CRRT use, day	1.13 (1.09, 1.16)	< 0.001
Duration of Mechanical Ventilator use, day	1.03 (1.02, 1.04)	< 0.001
Experience of CPR during hospitalization	1.70 (1.36, 2.13)	< 0.001
**Year of admission for ARDS**		
2015 (vs 2014)	1.13 (0.81, 1.58)	0.464
2016 (vs 2014)	1.05 (0.76, 1.44)	0.770
2017 (vs 2014)	1.12 (0.81, 1.56)	0.483
2018 (vs 2014)	1.30 (0.94, 1.78)	0.110
2019 (vs 2014)	1.57 (1.14, 2.15)	0.005

Hosmer Lemeshow test: Chi-square, 3.66, df = 8, *P* = 0.886.

*ARDS* acute respiratory distress syndrome, *ECMO* extracorporeal membrane oxygenation, *OR* odds ratio, *CI* confidence interval, *IM* internal medicine, *USD* United States Dollars, *NMB* neuromusclar blockade, *CRRT* continuous renal replacement therapy, *CPR* cardiopulmonary resuscitation.

**Figure 2:**
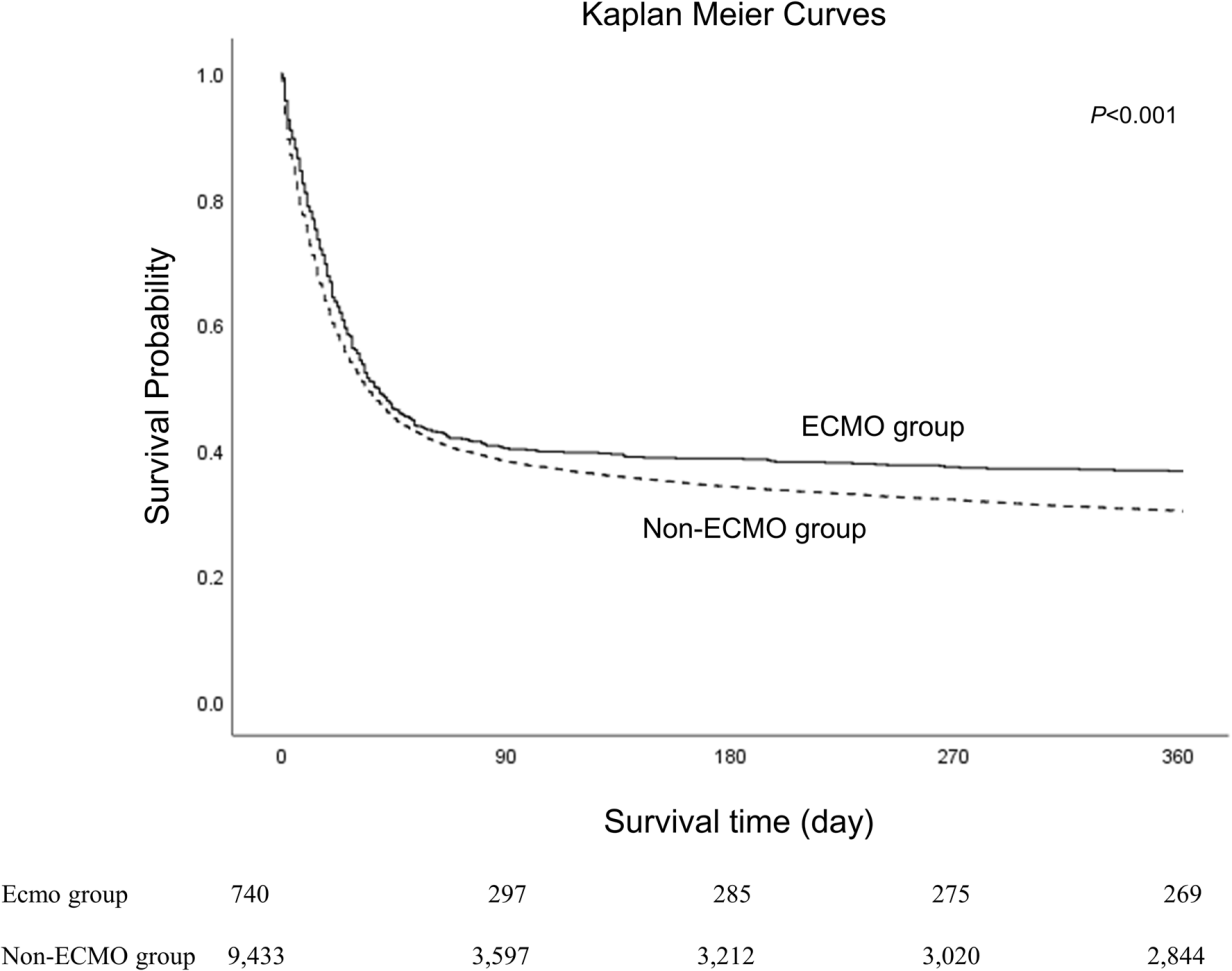
Kaplan–Meier curves of overall survival up to 365 days after ARDS diagnosis in ECMO and non-ECMO groups. ARDS. Acute respiratory distress syndrome, ECMO, extracorporeal membrane oxygenation.

Figure 2 shows the Kaplan–Meier curves of overall survival time up to 365 days after ARDS’ diagnosis in the ECMO and non-ECMO groups. The log-rank test showed that the median survival time was significantly longer in the ECMO group than in the non-ECMO (37.0 days; 95% CI: 29.6–44.4 in the ECMO group versus 34.0 days, 95% CI: 32.1–35.9 in the non-ECMO group; *P* < 0.001).

## Discussion

In this population-based cohort study using real-world data from South Korea, ECMO support was applied to patients with ARDS who were of younger age and had a higher annual income level. ECMO support was also performed more frequently in high case volume centers than in low case volume centers for patients with ARDS. Moreover, ECMO support was concomitant with other treatments, such as NMB use, CRRT support, and a longer duration of mechanical ventilation. Our results suggest that physical, socioeconomic, and clinicopathological conditions affect the application of ECMO support for patients with ARDS in South Korea.

In this study, the mean duration of ECMO support in patients with ARDS was 1.6 days (SD 2.8), which was relatively short compared to that observed in previous reports^15,16^. The difference might be due to the characteristics of patients who underwent ECMO support. First, patients without extremely severe acute respiratory failure who required a longer duration of ECMO support might have been excluded, based on the indications for ECMO support, due to an expected poor treatment outcome. Table 2 supports this assumption because age and CCI were lower in the ECMO group than in the non-ECMO group. Moreover, the total cost of hospitalization was significantly higher in the ECMO group than in the non-ECMO group, suggesting that patients with ARDS who were expected to recover with a better prognosis might receive active and invasive treatments, such as ECMO support, CRRT, and CPR, in South Korea. Therefore, the results should be interpreted carefully considering the shorter duration of ECMO support in patients with ARDS in this study.

The age of patients with ARDS is an important issue in the application of ECMO support. In South Korea, age is a major predictor of poorer survival outcomes in patients with ARDS who undergo ECMO support^17^. Mendiratta et al. reviewed elderly patients aged > 65 years who underwent ECMO support in the Extracorporeal Life Support Organization (ELSO) registry from 1990 to May 2013^18^. According to the ELSO database^18^, the number of elderly patients who underwent ECMO has increased in recent years, and Mendiratta et al. emphasized that old age should not be an absolute contraindication for the initiation of ECMO in patients with ARDS. Similar results were observed in the ELSO registry, and Lorusso et al. also insisted that old age should not be an absolute contraindication for applying ECMO in patients with cardiogenic shock^19^. Another study reported that hospital survival outcomes after ECMO support were similar in both elderly and young patients, suggesting that old age is not a contraindication^20^.

The results regarding annual income levels are also important in this study. We showed that patients with ARDS with lower annual income levels tended to avoid ECMO support compared to those with higher annual income levels. A previous systematic review reported that the total in-hospital cost for ECMO support ranged from 42,334 to 537,554 USD in 2013^21^. Another more recent review also reported that the costs of ECMO support ranged from USD 22,305 to USD 334,608 in 2019^22^. Although the South Korean government covers approximately two-thirds of the total medical expenses^23^, the financial burden might be a critical factor for applying ECMO support in patients with ARDS, as demonstrated in this study.

Additionally, we showed that patients with ARDS in South Korea received more ECMO support in high-volume centers than in low-volume centers. This might have been influenced by some examples concerning higher case volume centers in South Korea. First, a higher case volume center was associated with improved survival outcomes for patients with ARDS^24^. Second, a high case volume center was associated with better survival outcomes for patients who underwent ECMO support^25^. As ECMO support is a highly source-demanding procedure, outcome and quality of life after ECMO support might be better in selected and high-volume ECMO centers, which might affect the study results.

This study has several limitations. First, the severity of ARDS was not accurately assessed. For example, the PaO_2_/FiO_2_ ratio [Definition of ARDS: relation of the patient's oxygen in arterial blood (PaO_2_) to the fraction of oxygen in the inspired air (FiO_2_)] and Acute Physiology and Chronic Health Evaluation II scores were not included in this study for the adjustment of patients with ARDS. Second, there might be missing cases of individual underlying diseases using registered ICD-10 codes, which were used to calculate the CCI. Third, an important treatment option, such as prone positioning, was not considered in this study because the prescription code of prone positioning did not exist in the NHIS database. Lastly, some important covariates, such as smoking history, pulmonary function test results, and body mass index, were not adjusted because the NHIS database did not contain these data.

In conclusion, real-world data from South Korea showed that ECMO support was applied to patients with ARDS who were of younger age and had a higher annual income level. ECMO support was also performed more frequently in high case volume centers than in low case volume centers for patients with ARDS. In addition, ECMO support was used concomitantly with other treatments such as NMB use, CRRT support, and a longer duration of mechanical ventilation. Our results suggest that physical, socioeconomic, and clinicopathological conditions affect the application of ECMO support in patients with ARDS in South Korea. Moreover, we showed that ECMO support is a highly source-demanding procedure that requires treatment in selected and high-volume ECMO centers.

## Supplementary Information

Supplementary Information.

## Data Availability

Data will be available upon reasonable request to corresponding author.
